# Exploring the gender gap in the Spanish Wikipedia: Differences in engagement and editing practices

**DOI:** 10.1371/journal.pone.0246702

**Published:** 2021-02-23

**Authors:** Julià Minguillón, Julio Meneses, Eduard Aibar, Núria Ferran-Ferrer, Sergi Fàbregues

**Affiliations:** 1 Faculty of Computer Science, Multimedia and Telecommunications, Universitat Oberta de Catalunya, Barcelona, Spain; 2 Faculty of Psychology and Education Sciences, Universitat Oberta de Catalunya, Barcelona, Spain; 3 Faculty of Arts and Humanities, Universitat Oberta de Catalunya, Barcelona, Spain; 4 Faculty of Communication and Information Universitat Oberta de Catalunya Barcelona, Barcelona, Spain; Utrecht University, NETHERLANDS

## Abstract

Wikipedia’s significant gender bias is widely acknowledged. In this paper we analyze the Spanish Wikipedia with the aim of estimating the percentage of women editors and measuring their engagement and editing practices with respect to their men counterparts. To identify the gender of Wikipedia registered users, we analyzed both the information contained in their user profile and the information provided by users about themselves on their personal user pages. Using our own coding procedure, it is possible to identify a greater number of women than by relying only on the gender reported in their user profile. Combining both methods, our results show that the percentage of women is small, a meagre 11.6% of all analyzed editors, though there is still a significant percentage of users whose gender cannot be determined by either method. Men outnumber women in all Wikipedia namespaces in a ratio that is always equal to or greater than 3:1. This fact can be partially explained by the lesser persistence of women editors, who tend to leave Wikipedia much more quickly. There is, however, a small group of veteran women editors who, in some cases, surpass men editors in terms of their editing practices and participation in different Wikipedia namespaces.

## 1. Introduction

Wikipedia is without a doubt one of the largest human collaborative efforts ever undertaken. With the aim of becoming “the sum of all knowledge”, as stated in Wikipedia’s vision [1], it was launched on January 15, 2001, and today contains more than 40 million articles in 301 different languages [2]. It is ranked by Alexa as the fifth most popular website, usually appearing in the first page of results generated by search engines. The Spanish Wikipedia, the focus of this paper, is the ninth largest Wikipedia, with more than 1,580,000 pages, and the fourth according to number of registered editors, about 17,000 [3].

The importance of Wikipedia as the main source of information for most internet users is unquestionable. The site generates more than half a billion page views every day. Many people search for answers to so-called ‘popular culture’ questions: celebrities, music, films, TV series, sports, and so on. However, Wikipedia has also been acknowledged as the most important source of scientific and technical information for most citizens, including students at all levels of education. Wikipedia is currently the most important channel for the public communication of scientific knowledge, and scientists and scholars are increasingly aware of this fact [4, 5]. In higher education, both faculty and students are using Wikipedia as a valuable resource [6, 7], and the site is also becoming a gateway to open access journals [8].

Despite its alleged neutrality, which is in fact embedded in its second basic pillar [9], Wikipedia has been subject to the same biases as any other source of knowledge and information. A number of authors [10–13] have highlighted the fact that Wikipedia suffers from a severe and persistent gender bias, in terms of both content (articles about men outnumber those about women) and community (most editors are men). In this paper, we focus on the lower participation of women relative to men in Wikipedia, a topic that has received less attention than content bias. The Wikimedia Foundation itself is very much aware of the problem [14, 15] and has launched a number of different actions and projects to address the issue [16], with limited success.

With regard to users’ editing practices, albeit difficult to estimate and usually limited to the English Wikipedia, it has been reported that the percentage of women editors is between 10 and 15% or even lower [12, 17]. Since these data are derived from surveys, however, some authors suggest the actual rate could be higher [18]. The general feeling is that the Wikimedia Foundation “completely failed” to meet its goal of raising the number of women participants to 25% by 2015. As already stated, studies have tended to focus on the English Wikipedia [19], though a few have addressed Wikipedia in other languages, such as German [20] or Greek [21]. In Wikipedia in grammatically gendered languages (such as Spanish), registered users have the option to exhibit their gender in their profile. In the Spanish Wikipedia, *Usuario* (a masculine word in Spanish for user) is displayed by default on the personal page; where a user explicitly identifies herself as a woman, *Usuaria* (feminine) is shown instead. However, as Massa and Zelenkauskaite [22] have shown, only a small percentage of editors decide to include their gender in their profile, especially during the creation of a new user profile.

Using a five layer model, Lir [23] identified five factors preventing women from engaging in Wikipedia: negative site reputation, anonymity, fear, alienation, and rejection. According to this author, the small percentage of women editors could be explained by the low interest of women in engaging in online environments where men are the majority, as they perceive these spaces as hostile masculine territories [23–26]. Another possible explanation could be that women editors experience such hostility more acutely and hence drop out more quickly [23, 27, 28]. Nevertheless, it could also be possible that some women conceal their gender behind a neutral profile that discloses no personal information, and that the true percentage of women on Wikipedia is therefore higher. There are several possible reasons for this. For instance, Forte et al. [29] found that harassment and intimidation were the most mentioned concerns on Wikipedia, especially among women editors. Menking and Erickson [30] describe “wikistress” generated by conflict while editing as one of the principal reasons for dropping out or reducing their engagement in editing practices. As stated by Shane-Simpson and Gillespie-Lynch [31], anonymous editors are usually perceived as men, so the high proportion of anonymous editors contributing to Wikipedia may exacerbate the gender gap by creating the illusion (or perhaps the reality) of a male-dominated, overly critical, hostile editing environment.

It would therefore be of interest to determine the number of women editors beyond those that explicitly state their gender. This would allow us to better analyze editing practices according to editor’s gender and, thus provide an accurate quantification of the contribution of women editors on Wikipedia. There are three different ways to measure the presence of women editors in Wikipedia in the literature. The first is by analyzing Wikipedia editor profile metadata, which provides three options: neutral gender (actually, unspecified), woman, and man [17]. In the case of grammatically gendered languages such as Spanish, this also means that the Wikipedia personal page and its title change from the default *Usuario* (masculine) to *Usuaria* (feminine), thus explicitly identifying these users as women. Unfortunately, if they do not specify their gender, they appear as *Usuario*, thus inflating the perceived number of men editors. Accordingly, the decision of a user to explicitly declare their gender can be automatically gathered through MediaWiki API. Obviously, this only works for editors who choose to identify themselves as women or men, a choice that seems to be made by only a small percentage of the total number of editors [22], as discussed earlier. The second way to estimate the number of women on Wikipedia is using surveys [32], though some authors point out that surveys carried out by the Wikimedia Foundation may be biased [18]. Most of the surveys are completely anonymous, and both methods for estimating the percentage of women on Wikipedia are independent and unrelated; therefore, it is impossible to combine them and obtain a more accurate estimation. Finally, other authors have shown that it is possible to use off-the-shelf machine learning algorithms to reveal undisclosed personal traits–such as gender, religion or education–from editor engagement [33], but with limited accuracy.

As part of an interdisciplinary project on gender and Wikipedia, we propose to use a complementary method to that based on Wikipedia editor profile metadata. Our first goal is to estimate the number of editors by gender, by means of analyzing the content of their personal Wikipedia user page, using the personal information disclosed by Wikipedia editors about themselves [34]. The two procedures are complementary and can therefore be combined to improve the estimation of the number of women and men editors and explore differences in editing behavior. Next, we will use gender as the grouping variable to analyze possible differences in editing practices and engagement.

Our research questions address women’s active participation as editors and possible differences in both editing practices and content preferences, as follows:

- RQ1: How many women are among the active editors in the Spanish Wikipedia?
- RQ1a: What is the percentage of women among the active editors?
- RQ1b: What is the convergence between our coding procedure and the data provided by MediaWiki API?
- RQ1c: Are there differences between editors when it comes to how they disclose their gender on Wikipedia?
- RQ2: Do women and men show different patterns in their editing practices?
- RQ2a: Are there differences between men and women in terms of the intensity of their engagement as editors?
- RQ2b: Do men and women participate in a comparable way in the different Wikipedia namespaces?
- RQ2c: Do men and women continue as editors for similar periods of time?
- RQ3: Do women and men show similar content preferences in their editing practices?
- RQ3a: In terms of page content, are the edits of men and women distributed in a comparable way?
- RQ3b: Are there pages or groups of pages where women are clearly underrepresented or overrepresented?
- RQ4: Can we discriminate between men and women according to their editing practices?

## 2. Materials and methods

In this section we describe the dataset and the sampling strategy used to obtain the user profiles analyzed in this paper, the coding process used to determine gender according to the information revealed in the personal user page (if any), the measures that define users’ editing practices and engagement, and the analytical tools and tests used to determine differences in editing practices between users identified as men or women.

### 2.1 Dataset

To determine the gender of the user profiles, we used a Spanish Wikipedia dump (dated October 1, 2017) containing 90,827,797 page edits that allowed us to identify 963,591 registered editors (that is, discarding MAC, IP, and bot editions). Following Lam et al. [12], we focused on active editors (i.e., editors with at least 50 edits, who were active during the previous five years and therefore have made at least one edit since 01/01/2012). In the Spanish Wikipedia, this amounts to 28,763 editors. Finally, since we were interested in analyzing the information that editors disclose about themselves via their personal page, we filtered out editors who had not developed their own personal page, leaving a total of 13,210 editors. We described this population as “active editors in the Spanish Wikipedia”.

In the next step, the 13,210 editors were randomly sampled. Because of the power-law distribution of online participation, we considered that a simple random sample would contain mostly low-activity editors. Thus, we stratified our sample in order to equally capture all levels of engagement, according to two dimensions: number of edits and size of personal page. For each dimension, we generated four strata according to their quartiles, obtaining similar size partitions. Table 1 shows the number of elements (i.e., user profiles) in each stratum (N_i,_) and the number of randomly selected elements (NS_i,J_) in each stratum. To ensure that the user profiles were randomly assigned to the coders, we randomly selected three or four elements according to the available number of elements from each stratum. This procedure allowed us to generate a “package” containing up to 55 user profiles that we assigned to the coders. A total of 103 packages were generated, totaling 5,651 user profiles to be codified.

**Table 1:** Number of user profiles available / sampled in each stratum. Sampling is determined by the number of edits and the size of user page (in Bytes).

	Size of user page (in Bytes)				
Number of edits	1–186	187–705	706–2148	> = 2149	Total
50–93	1,277 / 412	1,074 / 412	664 / 309	297 / 295	3,312 / 1,428
94–222	974 / 412	994 / 412	841 / 309	493 / 309	3,302 / 1,442
223–848	647 / 309	752 / 309	978 / 412	917 / 412	3,294 / 1,442
> = 849	412 / 309	477 / 309	817 / 309	1,596 / 412	3,302 / 1,339
Total	3,310 / 1,442	3,297 / 1,442	3,300 / 1,339	3,303 / 1,428	13,210 / 5,651

At a 99% confidence level, this sampling method allows us to assume a maximum error of 5% for each stratum and 1.5% for the whole population. Accordingly, a total of 5,651 editor personal pages were analyzed, representing 42.8% of the editors under scrutiny. Table 2 shows the computed weight for the elements in each stratum analyzed in this study.

**Table 2:** Sampling weights for each stratum, determined by the number of edits and the size of user page (in Bytes).

	Size of user page (in Bytes)			
Number of edits	1–186	187–705	706–2148	> = 2149
50–93	1.3259	1.1151	0.9192	0.4307
94–222	1.0113	1.0321	1.1643	0.6825
223–848	0.8957	1.0411	1.0155	0.9521
> = 849	0.5704	0.6604	1.1311	1.6571

### 2.2 Coding

According to Wikipedia, user pages are administration pages that are useful for organizing and aiding the personal contribution of users on Wikipedia, as well as facilitating interaction and sharing between them. User pages are mainly conceived for interpersonal discussion, notices, testing, and writing drafts. In addition, users can decide whether to include biographical and personal content, the main focus of our analysis. In order to determine whether to attribute a user profile to a woman or a man, we designed a coding procedure that allowed us to determine the editors’ gender according to the information they disclosed on their user page. To do so, we used the following step-by-step procedure based on a set of simple rules:

- First, we checked the availability of the user profile, as some Wikipedia profiles may be empty, removed or locked, due to vandalism, plagiarism or some other reason. These profiles could not be codified and therefore were discarded.
- Where user profiles contained information and/or user boxes (small colored boxes designed to appear on a user page merely to communicate certain attributes reported by the user), we determined the editor’s gender and classified them as man, woman or unknown using the information presented on his or her personal user page:
- If the user explicitly provided information about his or her gender, we used this information to classify the user. Taking advantage of the grammatical gender encoded in the Spanish language, we used expressions such as *soy abogada* (“I’m a [woman] lawyer”) to identify the user as a woman. Additionally, in the presence of infoboxes (Wikipedia fixed-format tables) related to gender, we used them as a clear expression of the user’s gender.
- If the user provided their real name, it was also possible to determine his or her gender, given that most names in Spanish are indicative of gender and can easily be classified as a man or a woman. In case of doubt (i.e., the name is ambiguous in terms of gender and could equally be used by women and men), we did not apply this rule. On the other hand, given that women are less likely to reveal their gender through their chosen username [35], we did not use this information.
- If the user provided no explicit information about their gender, but this could be inferred from the use of grammatical gender in their self-descriptions, we used this information to identify them as a woman or a man user. For instance, we determined that a user was a woman if she wrote *Estoy interesada en*… (“I am interested in…”), since *interesada* is the feminine form of the participle “interested” in Spanish.
- Finally, if the above was not sufficient to determine gender, we classified the user as a user of unknown gender. For the sake of clarity, we refer to these users as “unknown”.

To ensure coding reliability, two researchers each independently coded two packages of user profiles, which contained 55 profiles per package. Given the cardinal nature of the users’ gender variable (i.e. man, woman, or unknown), we computed Krippendorff’s alpha [36] to determine inter-coder agreement, showing a high reliability with a K-alpha of 0.9635. In the next step, we trained a group of 39 coders, who coded the 5,651 profiles sampled in the study. We carried out the training in two sessions. In the first session, we presented the study (including the main aims and rationale of the study, and how Wikipedia works), explained the coding procedures, and described the content and structure of the coding book. In the second session, we assigned one package of user profiles to each coder and carried out individual one-on-one training with them. The training involved coding 50% of the assigned package and sending back the results of the coding to the members of research team, who checked for accuracy and discussed any discrepancies. Then, the coders coded the remaining 50% of their assigned user profile package to ensure the quality of the process. Once the two training sessions were completed, the coders coded a second package of user profiles that was individually assigned to them. During this process, the coders were in constant communication with the members of research team, who assisted them in case of uncertainty. Once the coding was completed, the final results were checked for accuracy by the members of research team.

### 2.3 Measures

To determine differences in editing practices, we used gender as the grouping variable, which takes three possible values: man, woman or unknown. Gender was determined by combining the gender specified in user’s profile (extracted through MediaWiki API [12]) with the gender extracted from the aforementioned content coding, using a simple majority voting scheme, with an “Unknown” class (that is, unspecified). Let us suppose that C_MAN_ is the gender computed by our manual content coding and C_API_ is the gender extracted through the MediaWiki API. Then:

- If C_MAN_ = C_API_ then class = C_MAN_
- If C_MAN_ = Unknown, and C_API_! = Unknown, then class = C_API_
- If C_MAN_! = Unknown, and C_API_ = Unknown, then class = C_MAN_
- Otherwise, raise an error as both procedures do not agree.

Editing practices and engagement were characterized according to the following attributes: participation (both strength and variety), lifespan, and content preferences [12, 33, 37].

Participation in different Wikipedia namespaces is a measure of their level and type of engagement [49]. Wikipedia divides its content into different namespaces, according to their purpose within the system, and each namespace has its own discussion pages designated for communication and coordination activities. For instance, the namespace named Wikipedia is used for administration pages with information or discussions about Wikipedia itself. Participation was measured using the number of edits in each namespace, namely, edits to content pages, personal user pages, talk pages, talk pages of other users’ personal pages, Wikipedia administration pages, and other namespaces. This is a measure of the total editing amount of effort carried out by each editor. Variety of participation was measured through the number of different pages edited by an editor, the average number of edits per page and the number of new pages started from scratch (i.e., created). This is a measure of how focused editors are across all their editing practices.

Regarding the lifespan of participation, we measured the number of days between the first and last edition, the number of active days (that is, days with at least one edition), the percentage of edits per active day, and the number of days since last edition. This latter information will be used as a measure of editors’ disengagement (i.e., to determine if an editor can be considered a dropout).

Content preferences were measured as the number of edits in special categories and groups of pages that we consider relevant to our purposes. We followed the procedure described by Lam et al. [12] using a different set of categories to those used for the English Wikipedia, given the particularities of the categories in the Spanish Wikipedia. Several authors have stated that Wikipedia’s categories are more of a folksonomy than a true taxonomy [37–39], and cannot be completely relied upon to organize and navigate through its content. Furthermore, Wikipedia’s categories have tended to be more stable at the bottom (i.e., the category terms on Wikipedia pages do not change over time) than at the top level [40], making top-level categories less reliable because they are occasionally reorganized. Currently, the Spanish Wikipedia defines five top-level categories, namely, Science, Humanities, Nature, People, and Society. However, since significant overlap exists between these top categories (for instance, Society includes all pages in People), we did not use them for grouping pages. Instead, to avoid overlap, we only considered those pages that were included in only one of these five top categories, discarding those belonging to two or more categories. Regarding individual pages, we only analyzed those with the largest proportion of editors identified as women and those related to WikiProjects that had a greater focus on gender issues. Since they serve as a starting point for becoming a Wikipedia editor [28, 41], WikiProjects are important coordination mechanisms for experienced editors as well as for newcomers.

### 2.4 Analysis

First, we used a Chi-squared test to measure differences in proportions among groups (i.e., by gender) with the aim of determining which method (API or manual coding) is more effective to identify the gender of editors. Then, in order to determine if there are statistically significant differences in editing practices among the three groups (i.e., men, women and unknown), we used a non-parametric Kruskal-Wallis H test, followed by Cohen’s d effect size, and complementary non-parametric post-hoc Dunn’s test with the Benjamini-Hochberg correction [38], as we have more than two classes. Finally, a Chi-squared goodness-of-fit test was also used to determine differences among groups when comparing the proportion of identified men, women and unknown users editing a page or group of pages with respect to the expected proportions.

## 3. Results

Our first result emerged from our manual coding procedure in answer to our first research question (RQ1a). We were able to code 4,746 of 5,651 personal pages, after excluding empty, removed, and locked user pages. Of these 4,746 personal pages, 2,029 were attributed to men (42.8%), 295 were women (6.2%), and 2,422 could not be attributed and therefore remained “unknown” (51.0%). We recalculated the sampling weights in Table 2 in order to account for the uncoded profiles, as shown in Table 3.

**Table 3:** Sampling weights for each stratum, determined by the number of edits and the size of user page (in Bytes), for the 4,746 coded profiles.

	Size of user page (in Bytes)			
Number of edits	1–186	187–705	706–2148	> = 2149
50–93	1.8650	1.0571	0.8005	0.4120
94–222	1.2913	0.9975	1.0277	0.5944
223–848	1.2044	0.9824	0.9150	0.8320
> = 849	0.8656	0.6617	1.0052	1.4817

Taking into account these corrected weights, and assuming a maximum error of 1.5%, our first estimation of the editors’ gender (according to our coding procedure) allows us to conclude that, among the editors of the Spanish Wikipedia, 6.1% are identified as women and 41.1% are identified as men, while the gender of the remaining 52.8% is unknown. If we take into account only user profiles attributable to women and men, those identified as women will represent 12.9% of the total number of editors. This figure is close to results obtained by comparable studies of the English Wikipedia, which have estimated the proportion of men editors at between 84 and 91% [10, 12, 18].

**Fig 1:**
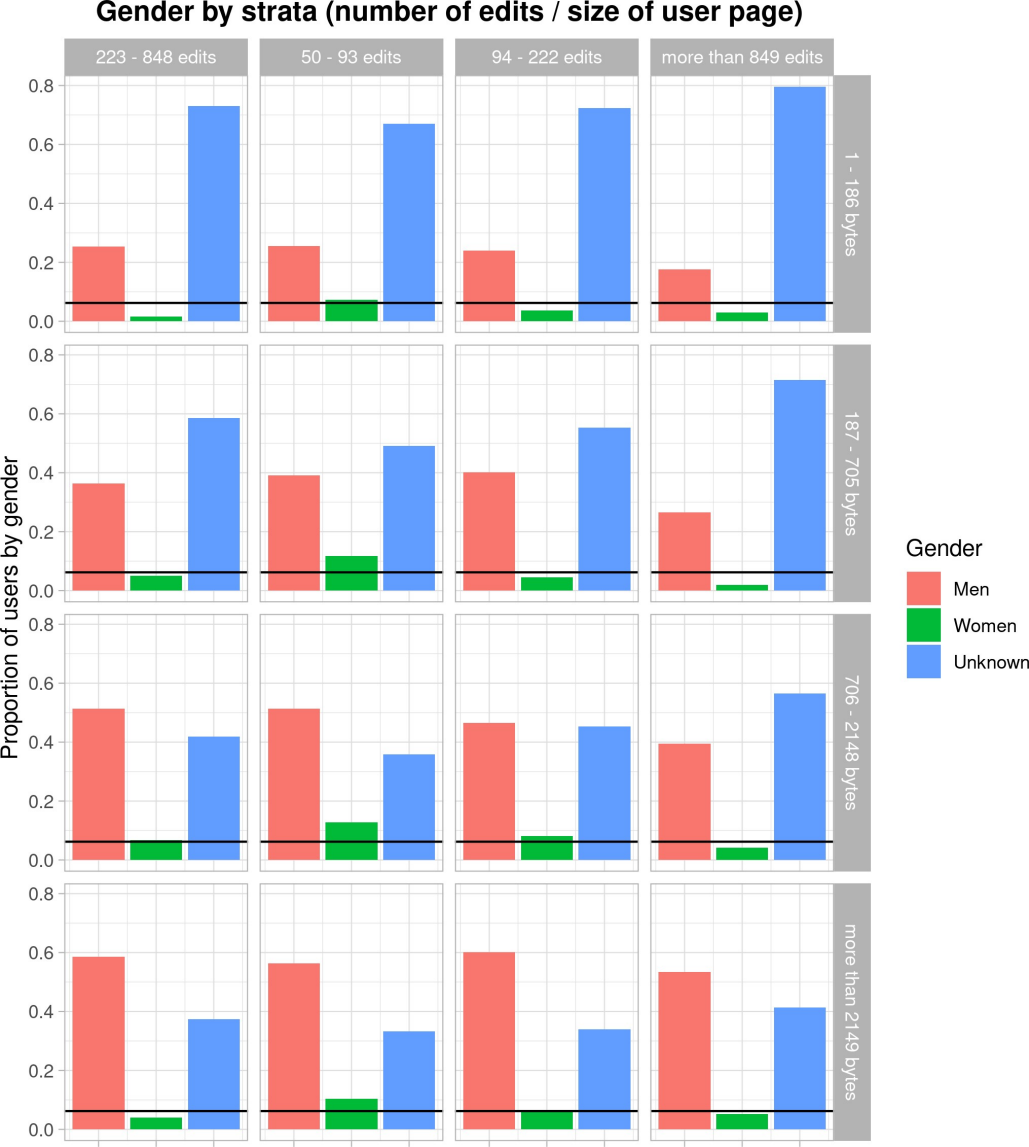
Distribution of gender by strata. For each stratum, the proportion of users identified as men, women or unknown are shown. The horizontal line represents the average 6.2% of users identified as women by our coding.

Fig 1 shows the distribution of men, women, and unknown user profiles across the stratum. Notice that these are clearly more present in the stratum “50–93 edits” (i.e., 10.8% of the users are identified as women) and less present in the stratum “849 edits or more” (3.8%). On the other hand, as expected, the bigger the user page, the more information is available to determine the editor’s gender and, therefore, the lower the percentage of unknown users on average.

### 3.1 Use of Wikipedia’s gender setting

As we have discussed previously, editors can specify their gender in their account’s preference settings. The gender setting is public information that can be extracted by means of MediaWiki API [12]. We used this method to extract gender for the 4,746 profiles previously coded. Then, we combined our coding method with gender setting using the simple majority voting scheme previously defined.

Table 4 shows the combination of both coding methods. For instance, there are 172 women identified by our coding method that did not specify their gender in their Wikipedia user profile. Notice there are no discrepancies between the two methods (i.e., the two zeros in Table 4), which is a good indicator that our coding method is adequate and that editors are presenting their gender in the same way via both methods. This answers RQ1b: What is the convergence between our coding procedure and the data provided by MediaWiki API? We claim to have found no discrepancies between the two procedures, which can therefore be used in a complementary way.

**Table 4:** Editors’ gender obtained by combining extracted from MediaWiki API and our content coding for the 4,746 coded profiles.

	Gender determined by content coding			
Gender determined by MediaWiki API	Unknown	Men	Women	Total (API)
**Unspecified**	1,601	**1,131**	***172***	2,904
**Men**	**763**	**898**	0	1,661
**Women**	***58***	0	***123***	181
**Total (manual)**	2,422	2,029	295	4,746

According to Table 4, therefore, we can affirm the following:

- There are 2,792 users identified as men (1,131+763+898, in bold), comprising 58.8% of the total number of editors.
- There are 353 users identified as women (172+58+123, in bold and italics), comprising 7.4%.
- The other 1,601 users remain labeled as “unknown” (33.8%).

Once again, if we take into account only editors identified as women or men, women comprise 11.2% of the total number of editors. Finally, if we take into account the stratified sampling weights after normalization for generalization purposes, there is a slight increase in this figure, from 11.2% to 11.6%. From here on, we use this subset containing 4,746 editors coded as women, men, and unknown to analyze their differences.

Table 5 shows the percentage of men and women according to the method used to identify their gender. Regarding their preferences about gender disclosure, only 1,842 editors (38.8%) used Wikipedia’s gender setting to reveal their gender in their profile. Women represent 9.8% of these editors. Among the 2,029 editors identified as men through the coding process (i.e., editors who have disclosed sufficient information in their user profile to enable them to be identified as men), only 898 (44.3%) specify their gender using the gender setting. In the case of users identified as women, the proportion is 123 out of 295 (41.7%), showing no significant difference among genders (Chi-squared = 0.706, p = 0.4006). On the other hand, among the 2,422 user profiles we were not able to code because of an absence of any gender-specific information on their personal page, only 821 specify their gender in their user profile (33.9%), which is significantly different from users properly identified (Chi-squared = 16.489, p < 0.001). In other words, users who did not declare their gender using the gender setting were also less likely to disclose information about their gender in their profile. Among the 821 editors who disclosed their gender in their user profile, women represent only 7.1%, a smaller percentage than the 11.6% we previously found. It is important to note that, among women, almost half of the classified profiles come from the coding process (48.7%), a higher percentage than among men (40.5%). On the other hand, fewer women are uniquely classified by their profile settings (16.4%), compared to men (27.3%).

**Table 5:** Percentage of users identified as men and women according to the method used to identify them (MediaWiki API, content or both).

	Identified by MediaWiki API	Identified by content coding	Identified by both methods	Total
**Men**	763 (27.3%)	1,131 (40.5%)	898 (32.2%)	2,792
**Women**	58 (16.4%)	172 (48.7%)	123 (34.9%)	353
**Total**	821 (26.1%)	1,303 (41.4%)	1,021 (32.5%)	3,145

According to these results, we can answer RQ1c: Are there differences between editors when it comes to how they disclose their gender on Wikipedia? Use of Wikipedia’s gender setting–available via the MediaWiki API–is comparable among men and women, but women are less likely to disclose personal information in their personal profiles. On the other hand, users with an unknown gender through coding are also less likely to use the setting to disclose their gender, thus reinforcing the idea that some users might be reluctant to reveal their gender.

### 3.2 Gender differences in editing practices

In this section we analyze the differences between men, women, and unknown user profiles with respect to their editing practices. First, we compare their participation in the different Wikipedia namespaces.

Table 6 shows the differences between men, women, and unknown gender profiles with respect to their total number of edits. These analyses have been carried out for all editors (N = 4,746) and for those 1,109 in the most active stratum (369 with an unknown gender, 689 men, and 51 women). For each namespace, the median of the number of edits is shown for each gender, as well as a multiple test of these differences through the Kruskal-Wallis H test and Cohen’s d effect size, and complementary Dunn’s test to show differences among each possible pair of groups. The last row of Table 6 shows the percentage of edits corresponding to non-content pages, that is, administration, discussion and user pages.

**Table 6:** Median number of edits by gender in different Wikipedia namespaces, for all editors (upper line) and for those editors in the most active stratum (lower line). Significant differences among men (M) and women (W) are shown in bold.

	Unknown (U)	Men (M)	Women (W)	Kruskal-Wallis H test and Cohen’s d effect size	Dif. U-M	Dif. U-W	Dif. M-W
**Total edits**	220.47	231.50	131.67	48.36 (p < 0.001) d = 0.199	No	Yes	**Yes**
	2173.60	3335.20	4037.51	26.00 (p < 0.001) d = 0.298	Yes	Yes	No
**Content**	162.70	163.70	80.16	68.11 (p < 0.001) d = 0.238	No	Yes	**Yes**
	1590.66	2301.01	3183.55	19.58 (p < 0.001) d = 0.254	Yes	Yes	No
**User page**	8.42	13.82	15.45	77.93 (p < 0.001) d = 0.255	Yes	Yes	No
	30.16	87.42	97.79	33.85 (p < 0.001) d = 0.344	Yes	Yes	No
**Talk**	3.93	4.58	1.03	49.95 (p<0.001) d = 0.202	Yes	Yes	**Yes**
	40.00	50.26	51.86	6.95 (p = 0.03) d = 0.134	Yes	No	No
**User talk**	4.53	5.35	3.66	17.49 (p < 0.001) d = 0.114	Yes	No	**Yes**
	47.41	71.12	106.55	17.66 (p < 0.001) d = 0.240	Yes	Yes	No
**Wikipedia**	0.98	1.60	0.92	27.69 (p < 0.001) d = 0.148	Yes	No	**Yes**
	14.82	29.15	112.59	27.91 (p < 0.001) d = 0.310	Yes	Yes	**Yes**
**Percentage of edits not in content pages**	19.47%	22.22%	27.57%	38.85 (p < 0.001) d = 0.177	Yes	Yes	**Yes**
	16.90%	23.09%	26.40%	23.77 (p < 0.001) d = 0.283	Yes	Yes	No

Excluding the number of edits to their personal page, users who are identified as men show higher figures than those identified as women, regardless of the Wikipedia namespace in which they participate. However, if we consider only the editors belonging to the most active stratum, all these differences become irrelevant except for the case of the namespace named Wikipedia, where users identified as women clearly outperform those identified as men. On the other hand, users identified as women have a higher percentage of edits in non-content pages. For those users identified as women in the most active stratum, this percentage is not statistically significant. Unsurprisingly, unidentified users focus more on content than on discussion and administration pages.

Table 7 shows the number of different pages edited by gender, number of edits per page and new content pages created, measuring the depth and breadth of interest of editors when editing Wikipedia content pages. Once again, results for all editors and those in the most active group are shown.

**Table 7:** Median number of different pages (content) edited and created by gender, for all editors (upper line) and for those editors in the most active stratum (lower line). Significant differences among men (M) and women (W) are shown in bold.

	Unknown (U)	Men (M)	Women (W)	Kruskal-Wallis H test and Cohen’s d effect size	Dif. U-M	Dif. U-W	Dif. M-W
**Number of different pages**	78.77	66.80	28.41	87.86 (p < 0.001) d = 0.272	No	Yes	**Yes**
	630.57	804.54	1671.69	15.96 (p < 0.001) d = 0.226	Yes	Yes	**Yes**
**Edits per page**	2.96	3.18	4.05	25.63 (p < 0.001) d = 0.142	No	Yes	**Yes**
	3.71	4.26	3.89	17.49 (p < 0.001) d = 0.238	Yes	No	No
**New content pages**	3.33	3.57	2.00	16.37 (p < 0.001) d = 0.110	No	Yes	**Yes**
	37.04	38.52	61.32	4.54 (p = 0.1) d = 0.096	No	No	No

In this case, users identified as men show more diverse interests than those identified as women, as they create and edit a larger number of different pages. Users identified as women, for their part, prefer to focus on a smaller number of pages. Nevertheless, this is not true for the most active editors, where users identified as women are more active with respect to creating and editing different pages and all gender differences vanish or are even reversed.

Thus, RQ2a and RQ2b can now be answered. With respect to RQ2a (“Are there differences between men and women in terms of the intensity of their engagement as editors?”), the answer is yes, users identified as men are much more active than those identified as women, and they create and edit more different pages. However, this is not true for the most active editors, where all gender differences disappear or are even reversed. With respect to RQ2b (“Do men and women participate in a comparable way in the different Wikipedia namespaces?”), the answer is no, since there are significant differences across all Wikipedia namespaces (where users identified as men constantly outperform those identified as women). Once again, however, this is not true for the most active editors, who show no significant gender differences, except for the Wikipedia namespace (aimed at content about Wikipedia itself), where users identified as women are much more active than those identified as men.

In both cases, these differences could be partially explained by the fact that women withdraw from editing Wikipedia sooner than men. Table 8 shows the number of days between first and last edition, the percentage of days within this period with at least one edit (i.e., active days), the average number of edits per active day, and the percentage of editors that have not edited for at least 26 weeks (half a year) since their last contribution.

**Table 8:** Lifespan, percentage of active days, intensity of editor’s activity and percentage of active users by gender, for all editors (upper line) and for those editors in the most active stratum (lower line). Significant differences among men (M) and women (W) are shown in bold.

	Unknown (U)	Men (M)	Women (W)	Kruskal-Wallis H test and Cohen’s d effect size	Dif. U-M	Dif. U-W	Dif. M-W
**Lifespan**	1970.24	1885.38	786.92	126.36 (p < 0.001) d = 0.328	No	Yes	**Yes**
	2494.97	2723.28	2600.52	8.05 (p = 0.02) d = 0.155	Yes	No	No
**% of active days**	4.51%	4.02%	5.51%	12.12 (p < 0.001) d = 0.092	No	Yes	**Yes**
	15.31%	21.60%	21.21%	33.63 (p < 0.001) d = 0.359	Yes	Yes	No
**Edits per active day**	4.72	4.44	5.93	38.88 (p < 0.001) d = 0.177	Yes	Yes	**Yes**
	7.40	9.00	12.65	22.37 (p < 0.001) d = 0.286	Yes	Yes	**Yes**
**% dropout**	11.51%	5.61%	28.51%	170.87 (p < 0.001) d = 0.384	Yes	Yes	**Yes**
	0.41%	0.75%	2.91%	1.22 (p = 0.54) d = 0.055	No	No	No

In general, users identified as women are editing for shorter periods than those identified as men, though during these periods they edit more regularly and more intensely. However, this is not true for the most active editors, who show no gender differences, except in the number of edits per active day, where users identified as women clearly outperform those identified as men. Nevertheless, the most dramatic gender difference observed in Table 8 is in relation to dropout (defined as not having edited half a year since the last edition), which is notably higher for users identified as women. Even such users in the most active editor group identified as women show a higher dropout rate when compared to those identified as men or unknown.

**Fig 2:**
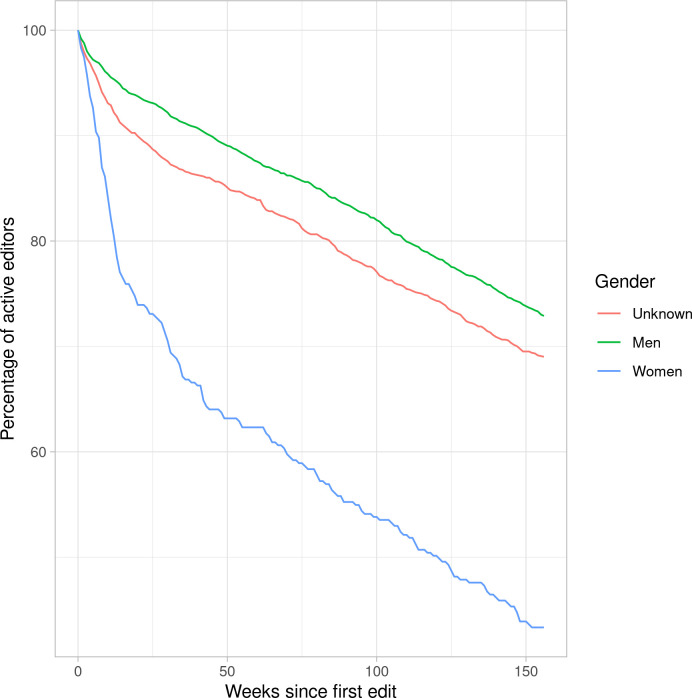
Percentage of editors active in the last 180 days since they last edit by gender.

Fig 2 shows the weekly percentage of editor activity by gender since their first edition, using a six-month window (180 days since their last edit). All three groups follow a similar decreasing rate after 16 weeks, but users identified as women have a larger dropout rate during this period. In fact, after 26 weeks, 95.2% of users identified as men are still active editors, in contrast to 76.4% of those identified as women.

This provides us with the answer to RQ2c (“Do men and women continue editing for similar periods of time?”). Evidently, users identified as women withdraw from editing Wikipedia much sooner than those identified as men, except for those in the group of most active editors. The dropout percentage is almost five times larger for users identified as women, an enormous and significant difference. In the case of the most active editors, dropout is significantly reduced, however, and there are no significant gender differences, though the dropout percentage remains higher for users identified as women.

### 3.3 Analysis of content areas

It is also possible to analyze whether men and women editors focus on different content areas. Table 9 shows the median number of edits to content pages included in only one of the five top categories available in the Spanish Wikipedia, by gender. For each gender group, the percentage of editors that edit in each category is shown. The proportion of women editing each category (with respect to all editors), and a Chi-squared goodness-of-fit test to determine whether they are editing significantly differently to expectation are also provided.

**Table 9:** Median number of edits and percentage of editors by gender editing the non-overlapping pages from the top five Spanish Wikipedia categories. Differences between women and the other groups are also shown.

	Number of edits			Women vs. Other		Women vs. Men	
Category Num. of pages	Unknown	Men	Women	%Women (7.4%)	Chi-squared test	%Women (11.6%)	Chi-squared test
**Science 39,433**	5.54 48.1%	5.48 54.7%	5.68 29.9%	4.4%	41.54 p < 0.001	6.5%	37.64 p < 0.001
**Humanities 30,424**	5.79 59.0%	6.06 61.8%	5.68 41.8%	6.9%	21.46 p < 0.001	10.3%	21.34 p < 0.001
**Nature 40,963**	4.50 36.3%	5.10 38.6%	4.52 23.7%	4.9%	19.19 p < 0.001	7.3%	18.98 p < 0.001
**People 8,601**	3.28 28.0%	4.74 33.2%	4.32 23.8%	5.8%	14.91 p < 0.001	8.4%	8.75 p < 0.001
**Society 149,463**	14.19 81.6%	16.56 83.3%	8.09 63.7%	5.9%	14.95 p < 0.001	8.9%	14.94 p < 0.001

This table reveals some interesting facts about content pages, although it is important to remember that we have not analyzed all Wikipedia content pages, but only those that belong to only one of the five top-level categories. First, users identified as women appear to be slightly less interested in editing pages related to Science and Nature (6.5% and 7.3%) than those identified as men. Worthy of mention is the fact that Science includes Technology, typically a male-dominated territory [25]. Nevertheless, the users identified as women who do edit Science pages are as active as their men counterparts with respect to the number of edits. Conversely, a larger proportion of users identified as women appear to be more interested in editing pages in the Society category (63.7%), though editors identified as men or unknown are still the majority in this category. The category with the largest percentage of users identified as women is Humanities (6.9%). Notice that all the percentages of editors identified as women are smaller than the expected 7.4% found in Table 4. This is consistent with two facts previously mentioned: with respect to their editing practices, women are slightly less interested in editing content pages (instead of discussion or administration pages), and they edit or create fewer different or new pages.

To extend our exploration of gender differences in content editing, we also analyzed individual pages where the proportion of women contributors is greater than expected. Given the immense number of pages to explore, we selected the most popular pages (in terms of participation). Accordingly, we selected only those content pages and their associated discussion pages with at least 50 editors, yielding a total of 1,403 pages. For each of these pages we then computed a Chi-squared goodness-of-fit test to compare the proportion of women editors with the expected probability of being a woman, excluding the unknown class.

Table 10 shows pages (in Spanish) on which the contribution of editors identified as women was greater than expected, that is, much higher than the 11.6% of editors identified as women. For the sake of clarity, the table shows only the first ten of the 58 pages selected (from a total of 1,403) where editors identified as women were found to be more numerous than expected (p < 0.05).

**Table 10:** Most popular pages with higher percentage of editors identified as women. For each page, the number of editors (N), number of edits (E) and average edits is shown by gender for each page.

	Number of editors (N)		Number of edits (E)		Edits per editor	
Page (in Spanish)	Men (N / %)	Women (N / %)	Men (E / %)	Women (E / %)	Men	Women
Thomas Alva Edison	35.74 72.7%	13.41 27.3%	102.40 69.9%	44.17 30.1%	2.87	3.29
Paulina Rubio	40.49 74.1%	14.13 25.9%	314.68 87.4%	45.22 12.6%	7.77	2.10
Feminismo	53.34 74.9%	17.84 25.1%	174.64 45.1%	212.33 54.9%	3.27	11.90
Estado de México	35.18 75.0%	11.73 25.0%	120.56 88.7%	15.38 11.3%	3.43	1.31
1984	43.08 75.0%	14.36 25.0%	82.90 45.1%	101.03 54.9%	1.92	7.04
Enrique Iglesias	35.57 75.5%	11.52 24.5%	76.23 60.2%	50.30 39.8%	2.14	4.37
Casi ángeles	39.20 75.9%	12.44 24.1%	723.05 64.0%	407.51 36.0%	18.45	32.76
Spam	29.60 76.5%	09.08 23.5%	87.39 84.0%	16.69 16.0%	2.95	1.84
Teclado (informática)	38.39 76.8%	11.60 23.2%	90.19 76.2%	28.16 23.8%	2.35	2.43
Mujer	43.78 76.8%	13.21 23.2%	137.20 64.1%	76.84 35.9%	3.13	5.82

As shown in Table 10, the ratio of users identified as men to those identified as women is approximately 3:1 on pages more frequently edited by women, though the number of participating editors and the number of edits show more variation. One possible explanation for this is that editors identified as men are everywhere on Wikipedia, regardless of page content, while editors identified as women tend to concentrate their edits in a reduced set of pages, such as “Feminism” (*Feminismo*), where they have relatively more presence and edit more than users identified as men (for instance, women are only the 25.1% of the editors that contribute to such page but they do the 54.9% of editions). Notice that in most pages, editors identified as women have, in average, more edits than editors identified as men. On the other hand, we found 54 pages where the percentage of editors identified as women was significantly smaller than expected (p < 0.05), among which there were seven pages that had never been edited by any woman. Four of these are related to soccer; the other three are “C” (the programming language), “Finland” (the country), and “José María Aznar” (the Spanish right-wing politician), which are apparently unrelated.

Thus, we are able to provide an answer to RQ3a (“In terms of page content, are the edits of men and women distributed in a comparable way?”). With respect to the presence of men and women across the most popular Wikipedia content pages (i.e., those that have received the largest number of edits), men editors appear to be present everywhere, though their average number of edits is quite variable between pages. Editors identified as women, on the other hand, appear to focus on a reduced set of pages, mainly because they make fewer edits.

These results could be attributed to personal preferences and the overwhelming presence of men editors rather than to a gender issue, except on a few pages that specifically address gender issues. When looking at the 10 articles with the highest rate of editors identified as women (Table 10), we found that two of them were ‘Feminism’ and ‘Woman’, while the rest did not show a clear common theme. For this reason, we considered that it would be interesting to carry out a specific analysis on pages related to gender issues. In this respect, we narrowed our analysis to other Wikipedia pages where women’s edits were greater than expected. We found *a priori* two interesting groups of pages, the first of which is composed of pages belonging to two WikiProjects, namely, “Women” and “Feminism”. The second group is composed of pages that contain “woman”, “women”, “gender”, “femin*”, and other gender-related words (or word fragments) in their title, yielding a total of 69 pages. In both cases, we included edits to content pages and their corresponding discussion namespaces. Table 11 shows the percentage of editors (for each one of the three groups) who contributed to each group of pages and the average number of edits by gender. A Kruskal-Wallis test is used to determine any gender differences between unknown, men, and women editors.

**Table 11:** Percentage of participation and mean number of edits in groups of pages considered to be related to gender issues. Significant differences among men (M) and women (W) are shown in bold.

	Unknown (U)	Men (M)	Women (W)	Kruskal-Wallis H test	Dif. U-M	Dif. U-W	Dif. M-W
WikiProjects	0.34% 0.0040	0.56% 0.0156	11.65% 8.3890	272.88 (p < 0.001)	No	Yes	**Yes**
Gender-related pages	4.49% 0.3243	6.03% 0.3228	10.00% 2.7359	9.88 (p = 0.01)	No	Yes	**Yes**

As expected, users identified as women were clearly more interested in editing these groups of pages, as a large percentage of them did so (with respect to the group they belong to), with a larger number of edits than users identified as men or unknown. This is especially true for WikiProjects related to gender issues, indicating that these seem to be a good way to attract women to Wikipedia. In answer to RQ3b (“Are there pages or groups of pages where women are clearly underrepresented or overrepresented?”), there are many pages on which women are clearly underrepresented, as most pages have a lower than expected percentage of editors identified as women; in some anecdotal cases, the percentage is even zero. This is especially true for pages with fewer than 50 editors. On the other hand, pages explicitly related to gender issues appear to attract a large percentage of editors identified as women, though those identified as men still outnumber or “surround” those identified as women in a ratio of 3:1 or higher.

### 3.4 Analysis of unknown profiles

Using the data about editors and their editing practices, we tried to measure the degree of separability between men and women, as points in an n-dimensional space characterized by the measured variables. As an exploratory analysis, we just wanted to determine whether the typical participation and engagement measures [12] are enough to discriminate among genders, as well as to make a reasonable prediction for the unknown editors. We used the following eight variables from Tables 4–6 to characterize editors: number of edits, number of different pages edited, number of pages created, lifespan in days, average number of edits per page, percentage of active days, and average number of edits per active day. As most of these variables are very skewed, we applied logarithms and then scaling to reduce their range to [0,1]. For computing distances between editors we used the Canberra distance, since it is less sensitive to extreme values [42], which were typical for the measured variables. Then, for each editor, we computed the nearest editor (i.e., the most “comparable” editor according to their editing practices on Wikipedia), and then we compared their genders. If men and women had different editing practices, they would be mainly surrounded by other users of the same gender. Table 12 summarizes the results of this analysis.

**Table 12:** Percentage of men and women if they were classified as the gender of their nearest neighbor, according to their editing practices.

	Predicted (i.e. nearest neighbor) gender	
Gender	Men	Women
Men	90.2%	9.8%
Women	79.6%	20.4%

Most users identified as men (90.2%) would be correctly classified using this procedure, while most users identified as women (79.6%) would not. Therefore, building classifiers that could help to discriminate between men and women by considering their editing practices is faced with two important issues. The first of these is the well-known gender bias that causes an unbalanced data set (almost 8:1 against women, according to our results). Secondly, the low degree of separability among women which makes all the typical balancing techniques useless, especially those based on oversampling the less populated class [43].

Finally, if we attempt to classify the 1,601 unknown editors according to their nearest editor (i.e., a man or a woman), we find that only 198 (12.4%) of those editors would be classified as women, a figure only slightly higher than the overall 11.6% found previously. In answer to RQ4 (“Can we discriminate between men and women according to their editing practices?”), we can just point out that editor’s gender cannot be determined according to their editing practices, as women are completely “surrounded” by men.

## 4. Discussion

Our analysis provides a detailed view of the editing gender gap in the Spanish Wikipedia by focusing not only on the number of men and women editors, but also on the number and type of their editing practices. Before addressing the implications of our results, we must discuss some of their limitations. First, we analyzed a dump, which is a static picture of Wikipedia at a specific moment in time. All the results described in the previous section, therefore, must be properly contextualized to that moment in particular. Second, we established a minimum of 50 edits as a threshold for analyzing editor activity, which is a typical setting for this kind of analysis [12]. However, this threshold may exclude novel women editors that participate in an edit-a-thon and have not yet reached this threshold. Our sampling was done according to the number of edits and the size of the users’ personal page, as these criteria are directly related to our goal, although other measures could have been also used as sampling criteria. Finally, we used a binary concept of gender, which is clearly an oversimplification and does not capture the possible differences among genders when revealing personal information. Nevertheless, we followed this approach because non-binary profiles cannot be determined neither by the user profile settings nor by gendered sentences extracted from editors’ personal pages.

The traditional method used to attribute editor gender using MediaWiki API enables the identification of a less than satisfactory 3.8% of women editors and 35.0% of men, while an overwhelming 61.2% remain unknown. As we have discussed, editors appear not to disclose their gender through the Wikipedia user profile [22]. In fact, Ferran-Ferrer et al. [44] found that some women editors were unaware of this possibility. Considering only profiles attributable to men or women, the percentage of women editors is 9.8%, a figure comparable to results obtained by studies on English Wikipedia [11, 12]. Our coding procedure allows us to attribute a gender to 49.0% of editors, only 38.8% of whom used the Wikipedia gender setting to reveal their gender. Therefore, our procedure provides a more accurate way of quantifying gender imbalance in Wikipedia, taking advantage of information disclosed by editors on their user pages. It is also remarkable that men are more likely than women to specify their gender in their profile (59.49% vs. 51.27%), whereas the percentage of men self-disclosing their gender via their personal page is smaller than that for women (71.12% and 83.33%, respectively).

As in previous studies [11, 12], we found that men editors drastically outnumber women editors. When combining both coding procedures, the percentage of active women editors on Spanish Wikipedia is 7.4%, increasing to 11.6% if only men and women (no unknown profiles) are considered. This makes Spanish Wikipedia a male-dominated territory and one that would plausibly be considered a misogynist space by women editors [14]. On the other hand, the large percentage of unknown profiles does nothing to help reduce this perception, as women perceive anonymous editors as more critical of their contributions [31].

With respect to the kinds of pages contributed to, our results show that women have higher percentages of edits on non-content pages than men, and less than them on content pages (i.e., Wikipedia articles). Since most non-content pages are aimed at interacting with other editors–particularly talk pages, where editors discuss shortcomings and possible improvements to Wikipedia articles–our findings seem to coincide with Lam et al.’s [12] assertion that, while men are frequently “contributors”, women tend to behave more as “collaborators”, adopting different roles [45]. This would be also consistent with the findings of Rizoiu et al. [33], who claimed that women tend to be marginally more interested in discussion and administration issues than in creating new content. Nevertheless, when considering only the most active editors, although women have a higher median number of edits in all namespaces, the differences between most active men and women are not statistically significant.

We also analyzed whether men and women editors have similar interests, using the top-level Wikipedia categories to group pages into disjoint sets, finding that women appear to be slightly less interested in editing pages about Science and Nature, but these top-level categories are too general and inaccurate, as already discussed. Our results are somewhat consistent with the findings of Lam et al. [12], but not with those described by Rizoiu et al. [33]. However, it is important to consider that Spanish and English categories do not necessarily include the same type of articles. Women editing in each category show a smaller median number of edits than men, except for pages belonging to the Nature category, where this number was slightly higher. Nevertheless, no significant gender differences were found with respect to the number of edits.

Regarding specific content pages, men editors always outnumber women in a ratio of 3:1 or higher. In fact, there is a long list of Wikipedia articles to which women contribute significantly less or even not at all. We were only able to find two groups of pages where women seem to be more active than men, which were two WikiProject pages (“Women” and “Feminism”) and another group of pages related to gender issues. These pages were edited by a tiny fraction of editors, however. Accordingly, women seem to be more interested than men in contributing to WikiProject pages focused on closing gender gaps.

However, when we concentrate our attention on the most active editors, gender differences among women and men editors vanish or are even reversed, with women more active than men in some cases. However, women editors are a clear minority. Women who become established Wikipedia editors achieve higher number of edits over time, yet our results also point to a clear difference between women and men in terms of Wikipedia engagement. Women withdraw from editing Wikipedia sooner than men, especially during the first weeks. Therefore, there is a lower retention rate among women than men, a finding consistent with Lam et al. [12]. Although edit-a-thons oriented toward introducing women to Wikipedia appear to be a good lure [28, 46–48], these friendly and supportive scenarios need to be more than sporadic events that disappear a few days later [19, 49]. Indeed, this is a well-known factor reported by women editors [44].

## 5. Conclusions

Our study is one of very few to provide a detailed analysis of the gender gap in the Spanish Wikipedia while adding to knowledge of Wikipedia in other languages. In contrast to other ways of estimating this gender bias, we have mainly analyzed the information disclosed by editors about themselves in their user profiles. Unsurprisingly, our research confirms the well-known fact that women editors, a mere 11.6% of all editors whose gender was identifiable, are hardly present as contributing editors. In tackling this issue, we have combined two methods of gender determination. The first of these relies on the user’s self-presented gender disclosure on their Wikipedia user profile, a method only used by 38.8% of the editors considered in our analysis. The second is a coding process that allows us to attribute gender according to information disclosed on editors’ personal pages. Our coding process helped us to identify the user’s gender in 49.0% of cases. As we have shown, this is particularly relevant for women editors, who appear less likely to disclose their gender using Wikipedia’s gender setting.

Generally speaking, women are not only a minority among Wikipedia’s editors, but their editing practices also appear to be less intense compared to men. In fact, Wikipedia is an immense space in which women may feel outnumbered by men (and indeed they are, according to our results and the available literature), even on gender-issue oriented WikiProject pages. This fact may incline women not to disclose their gender via the Wikipedia gender setting or self-presentation on their user page. However, we also found that women who achieve a certain level of edits over a long period become as active as men on Spanish Wikipedia, or even more so in terms of their editing practices. Perhaps, in addition to well-established initiatives such as edit-a-thons and WikiProjects designed to attract more women to Wikipedia, it would also be useful to make the involvement of this highly active group of women editors more visible. Increasing awareness of women’s successful experiences could be a good starting point to encourage other women to continue editing, and thus promote the long-term engagement of women on Wikipedia.

Based on the quantitative findings of this study, future research on this topic should focus on the reasons why women withdraw from editing in Wikipedia, a topic which is still largely unexplored. Further qualitative studies could provide a more fine-grained, in-depth understanding of the current gender gap in Wikipedia by, for instance, exploring the views of women on a framework for gender equality in this collaborative environment, or by describing their experiences as content generators in a highly male-dominated environment. Deeper analysis of the role of edit-a-thons is also needed for understanding the self-inclusion strategies that can enable more women to adopt and maintain a habit as regular editors over time.
